# Supplementary material for: The microbiome biomarkers of pregnant women’s vaginal area predict preterm prelabor rupture in Western China
Source: Front Cell Infect Microbiol. 2024 Oct 31;14:1471027. doi: 10.3389/fcimb.2024.1471027 (PMC11560878; doi:10.3389/fcimb.2024.1471027)
Supplement: Supplementary file 1 [file DataSheet1.zip › compare_1/Community/Pieplot/species/sample/P30.species.pieplot.pdf]

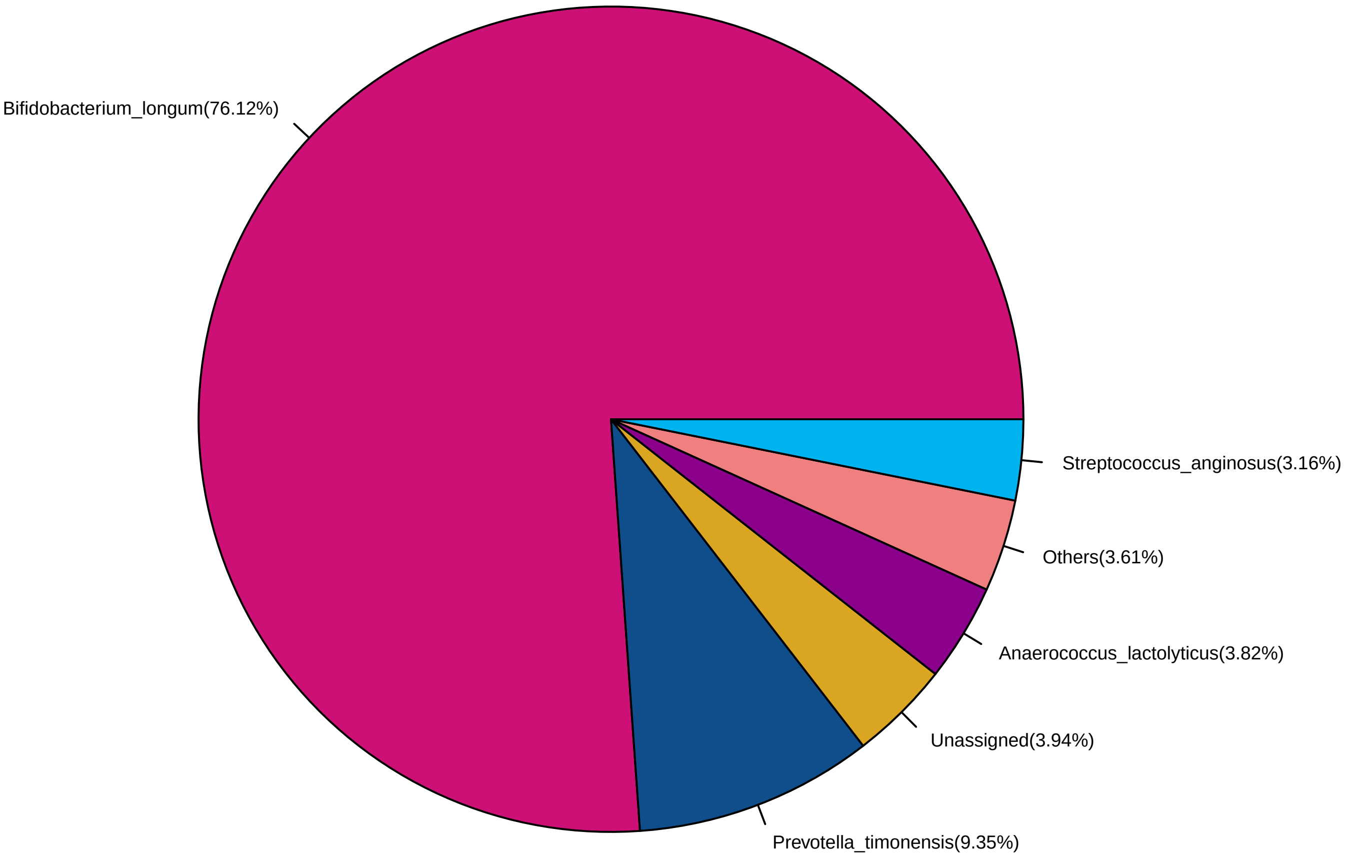

Bifidobacterium_longum(76.12%)
Streptococcus_anginosus(3.16%)
Others(3.61%)
Anaerococcus_lactolyticus(3.82%)
Unassigned(3.94%)
Prevotella_timonensis(9.35%)
